# Comparison between 1.5 and 3-Tesla MRI findings in Ménière’s disease

**DOI:** 10.3389/fneur.2024.1458227

**Published:** 2024-10-31

**Authors:** Juliana Antoniolli Duarte, Marcio Ricardo Taveira Garcia, Soraia Ale Souza, Rafael da Costa Monsanto, Maira de Oliveira Sarpi, Amy Juliano, Fernando Freitas Gananca

**Affiliations:** ^1^Federal University of São Paulo (UNIFESP), Otorhinolaryngology Department, São Paulo, Brazil; ^2^Diagnosis of the America S/A (DASA), Department of Radiology, São Paulo, Brazil; ^3^Medicine Faculty of the São Paulo, University of the State of São Paulo (FMUSP), Department of Pediatric Radiology, Minneapolis, MN, United States; ^4^University of Minnesota Health Sciences, University of Minnesota Medical Center, Minneapolis, MN, United States; ^5^Harvard Medical School, Department of Radiology at Massachusetts Eye and Ear, Cambridge, MA, United States

**Keywords:** Meniere disease, 3 Tesla MRI, hydrops, endolymphatic and perilymphatic space, endolymphatic hydrops

## Abstract

**Introduction:**

3 T-MRI of the inner ear has been used to identify the endolymphatic hydrops (EH) phenomenon, and less frequently 1.5 T-MRI. The aim of this study was to assess whether there was agreement between findings of EH at 1.5 T MRI and those obtained at 3.0 T MRI in patients clinically diagnosed with definite Meniere disease (MD).

**Methods:**

Cross-sectional, blinded study was conducted in a tertiary neurotology ambulatory practice. Thirty patients with clinical diagnosis of unilateral definite MD was included. Two MRI exams (1.5 T and 3.0 T) were performed for each patient and were evaluated by two examiners (E1, E2) who were blinded to the symptomatic ear. An analysis of intra-and inter-examiner agreement was performed. It was determined whether there was an association between MRI findings and disease duration, symptom severity, and MD clinical stage.

**Results:**

E1 found EH at 3 T-MRI in 26 (86.66%) patients and at 1.5 T-MRI in 25 (83.33%). E2 found EH in 25 (83.33%) patients in 3 T-MRI and in 22 (73.33%) at 1.5 T-MRI. The agreement between the examiners’ assessments in relation to the EH was high (0.844) for the 3 T MRI and substantial for the 1.5 T, both statistically significant. There was no statistically significant relationship between EH imaging findings and clinical disease severity and course.

**Discussion:**

1.5 T and 3.0 T MRI images agreed regarding the findings of absence or presence of cochlear hydrops (CH) and vestibular hydrops (VH). The degrees of CH and VH found at 3.0 T MRI in symptomatic ear were not associated with clinical aspects and the stage of disease.

## Introduction

Meniere’s disease (MD) is a multifactorial episodic clinical syndrome characterized by hearing and vestibular symptoms. The peak of incidence is between the third and seventh decades of life (1–3). The presence of endolymphatic hydrops (EH), described by Hallpike and Cairns, is the hallmark of the disease (4). Although the diagnosis of MD is mostly clinical, some cases present with atypical symptoms, and therefore complementary hearing and vestibular functions tests may be used. Recently, the use of delayed contrast image acquisition using magnetic resonance imaging (MRI) has been proposed to evaluate the presence of EH.

The function of MRI for the evaluation of EH is to provide *in vivo* anatomical information that before the 21st century was only possible in *postmortem* histopathological analysis. Since the initial description published by Nakashima et al., who demonstrated the borders of the endolymphatic space (ES) using 3D-FLAIR image sequence in a 3 T MRI following application of gadolinium contrast agent (GC) transtympanically (5), other authors have successfully demonstrated that 3 T MRI is able to detect the presence of EH using alternate methods, including intravenous (IV) contrast injections and other types of MR imaging sequences.

At present, many countries have carried out 3.0 T MRI enhancement of inner ear, but there are only three published studies on 1.5 T MRI, with a protocol dedicated to the detection of EH (6–8). Grieve et al., used inversion recovery (IR) sequences with three inversion times with intratympanic GC, observing HE in 11 patients of the 12 in the sample (6). Naganawa et al., used 1.5 T in the evaluation of 20 patients through the use of the HYDROPS protocol and use of IV GC, and observed EH in the 40 ears examined (7). However, these studies have as a limitation the small sample size, the patients had a diagnosis of unilateral or bilateral, possible or definite MD, and there was no comparison with the clinical characteristics of the patients (7). There is only one study to compare 1.5 T MRI and 3.0 T MRI that used GC transtympanic (8). Han et al., conducted a case–control study with 25 MD patients examined by 1.5 T MRI and 51 MD patients examined by 3.0 T MRI, both groups were injected with GC into bilateral tympanic membrane, and 3 dimensional-Fluid Attenuated Inversion Recovery (3D-FLAIR) MRI was performed 24 h later. Positive rate of EH was 96% in the 1.5 T group and 96.1% in the 3.0 T group and the authors conclude that 1.5 T and 3.0 T MRI have the same value and significance (7).

One of the advantages of using 1.5 T MRI instead of 3 T MRI is that 1.5 T equipment is more widely available, especially in developing countries (9). If the MRI exam is more accessible, it will be able to participated for routine of the complementary tool in MD, just like the functional exams of nystagmus and electrocochleography.

Although the feasibility of 1.5 T MRI to detect EH was demonstrated in a preliminary studies (6–8), whether those results are reproducible is yet to be fully determined. Therefore, the aim of this study was to assess whether there was agreement between the findings of EH at 1.5 T MRI and those obtained at 3.0 T MRI in patients with a clinical diagnosis of definite MD. To further explore the practicality and maneuverability of 1.5 T MRI inner ear GC in the diagnosis of inner ear membrane labyrinth hydrops and to further expand the use of 1.5 T MRI GC enhancement of inner ear in the world.

## Methods

We conducted a cross-sectional, blinded study, that included patients from the Neurotology outpatient clinics of two institutions. The study was approved by the Research Ethics Committee under the protocol number: 01741818.2.0000.5505. All patients signed the Informed Consent Form.

The study was blinded to the radiologists involved in the analysis of the MRI results, so only one medical researcher, had access to the patients’ clinical records, which were in password-protected electronic medical records. This ensured patient confidentiality and the protection of their data.

### Patients

We identified cases of definite MD based on the criteria proposed by the *American Academy Otolaryngology-Head and Neck Surgery* (AAO-HNS), 1995 (10) and currently by the *Bárány Society*, in 2015 (1), which are: two or more episodes of spontaneous vertigo lasting from 20 min to 12 h; audiometric documentation of sensorineural hearing loss (SNHL) in low to medium frequencies in one ear, defining the symptomatic ear (SE) on at least one of the occasions, which may have occurred before, during or after an episode of vertigo; presence of fluctuating auditory symptoms (hypoacusis, tinnitus, or aural fullness) in the SE and exclusion of other vestibular diagnoses. The ear with an increase in the bone conduction threshold of at least 30 dBHL in two contiguous frequencies below 2,000 Hz compared to the contralateral ear without HL was considered as SE. If multiple audiograms were performed, demonstration of recovery of low-frequency SNHL at some point was also considered for the diagnosis of definite MD (1).

We excluded patients who underwent invasive or surgical procedures (such as IT injection, endolymphatic sac decompression, neurectomy, and other previous middle and inner ear surgeries) and patients who had diabetes mellitus, hearing loss from other causes, inner ear malformations, nephropathy, and those with a known allergy to gadolinium and contraindications to MRI.

Thirty patients with clinical diagnosis of unilateral definite MD were included, totaling 30 SE and 30 asymptomatic ears (AE). All patients had at least one episode of vertigo within the last year. None of the patients had vestibular symptoms between the first and second MRIs.

### Clinical assessment

We analyzed the following clinical parameters: number of typical episodes in the last year; duration of illness; dizziness handicap inventory (DHI) score; previous treatments; and possible phenotypic characteristics of MD. Patients with MD were categorized from 1 to 4 according to their degree of hearing loss, as proposed by the AAO-HNS (10). We collected information regarding medications that patients were taking to treat MD. The phenotypical characteristics were evaluated using the classification proposed by Frejó et al.: (phenotype 1) sporadic and classic MD, characterized by no history of migraine and autoimmune disease (AD); (phenotype 2) MD defined by the presence of hearing loss, which precedes vertigo episodes for months or years (late MD) and without a history of migraine or AD; (phenotype 3) familial MD (the patient has at least 1 first-degree relative with MD); (phenotype 4) MD with migraine symptoms; and (phenotype 5) MD symptoms with a possible associated AD (11).

We classified the stage of MD in symptomatic ears based on the pure-tone average score at frequencies ranging from 500 Hz to 3 kHz: stage 1, ≤25 dBHL; stage 2, 26–40 dBHL; stage 3, 41–70 dBHL; and stage 4, >70 dBHL (10).

### Image acquisition

The two MRI exams were performed within an interval of 1 week, in random sequence. The protocol performed in both MRI exams consisted of two phases. In the first phase, T1-weighted, T2-weighted, FLAIR, and DWI sequences were performed following IV administration of the contrast agent Dotarem® (gadoteric acid 0.5 mmol/mol) at a dose of 0.2 mmol/kg of patient body mass; in the second phase or late phase, which was performed within 4 h after administration of the contrast medium, 3D-FLAIR sequence were acquired.

3 T MRI was performed on a GE unit, model 750 GEM with 94 channels. The parameters used for the late phase were: FOV 190 mm, slice thickness 0.8 mm, TR 6,000 ms, TI 1,509 ms, TE 177 ms, flip angle 120°, matrix 288 × 288, bandwidth 213 Hz/pixel, number of excitations 1, FLAIR sequence voxel 0.7 × 0.7 × 0.6, turbo factor 1.25, and acquisition time of 15 min.

1.5 T MRI was performed on a Philips unit, Ingenia Digital model with 32 channels. The parameters used for the late phase were: FOV 110 mm, slice thickness 0.8 mm, TR 7000, TI 2250, TE 290, *Flip Angle* 40°, matrix 124 × 122, *Bandwidth* 116.8 T/s, number of excitations 2, FLAIR sequence voxel 0.9 × 0.9 × 0.9, turbo factor 150, and acquisition time of 15 min.

### Image analysis

The benefit of the initial phase is detecting any abnormal enhancement in the labyrinth or along nerves and the morphology of the inner ear structures and the presence or absence and caliber of the vestibulocochlear nerve. The second phase allowed delineation of the ES (negative contrast) from the perilymphatic space (PS) (positive contrast) and detection of ES dilatation, the imaging feature of EH. The ear with cochlear hydrops (CH) and/or vestibular hydrops (VH) of any degree seen on MRI is defined as the ear with EH.

Two examiners, who were Head and Neck-specialized radiologists, analyzed the MRIs (blinded to the clinical SE). The parameters evaluated were the cochlear duct area, saccule area, and utricle area, and grading of the degree of CH and VH. These parameters were evaluated for each ear and on both MR exams. For the grading of the degree of EH at MRI, the classification by Bárath et al. (12) was used. EH was considered present when the cochlear duct, saccule, and/or utricle appeared distended (or abnormal) on MRI. In the axial 3D-FLAIR sequence, EH was graduated: grade 0 (absent), in the cochlea, when there was no MR displacement, and in the vestibule, when there was a separate visualization of the saccule and utricle; grade I, in the cochlea, when was a mild dilation of the cochlear duct, without complete effacement of the cochlear vestibular scale, in the vestibule, when was a distension of the saccule and/or utricle, with the perilymphatic space still visible around them in the periphery of the vestibule; grade II, in the cochlea, when the vestibular scale was uniformly obstructed by the cochlear duct, which was maximally distended, in the vestibule, when the entire vestibule was encompassed by the dilated ES, with the perilymphatic space no longer being visualized.

### Statistical analysis

EH findings were described quantitatively for each examiner, for each MRI and for each ear. Agreement analyses were performed for the following using the kappa coefficient (*κ*) with a 95% confidence interval: (1) for the EH degree between the two examiners and for each of the two MRI (intra-and inter-examiner); (2) for HE parameters by MRI in the SE for each examiner and for each MRI; (3) for the parameter of HE in the 3 T MRI and HE in the 1.5 T MRI for each of the examiners; and (4) for examiner 1, between the CH/VH grade and the clinical parameters, between the CV/VH grade and the MD stage, and between the CH/VH grade between SE and AE for each of the two MRI.

For the qualitative variables, frequencies and percentages were calculated and were evaluated using Pearson’s chi-square test. Quantitative variables were presented as mean, median, minimum, maximum, and standard deviation and were evaluated using the nonparametric Mann–Whitney test. The significance level considered was 5% for both hypothesis tests. Analyses were performed using SPSS v.25 for Windows statistical software.
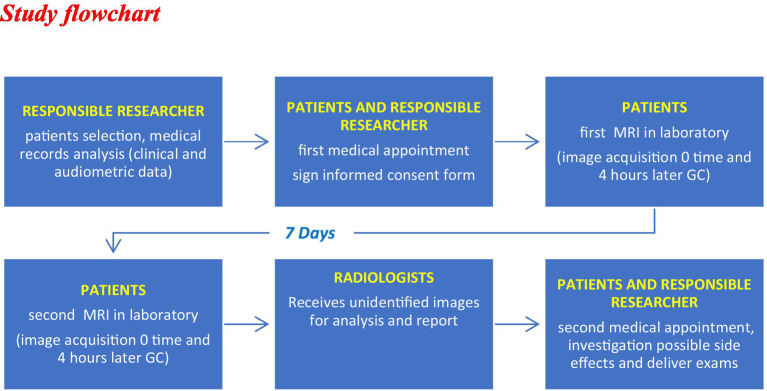


## Results

### Clinical assessment

Seventeen of the 30 patients (57%) were female and the mean age was of 49.6 years. The disease duration was on average 7.2 years, and the average number of vertigo episodes in the last year was 4. The mean DHI score was 43.8. Regarding treatment, 14 (46.7%) were taking betahistine in isolation, while flunarizine and meclizine were used by 8 (26.6%). Six (20%) were using venlafaxine (2 in isolation and the 4 others in associated with betahistine), and the remaining 2 (6.6%) were taking oral steroids in association with betahistine. Regarding clinical staging, most patients were classified as grade 3 (76.7%). Phenotypically, none of the patients were classified as phenotype 3. There were 13 cases classified as phenotype 1, 8 cases classified as phenotype 4; 6 as phenotype 2, and 2 as phenotype 5. Although none of the patients with phenotype 5 had confirmed autoimmune diseases, the characteristics of their MD (onset at an early age and dramatic response with the use of oral steroids) indicated as a possible autoimmune etiology (Table 1).

**Table 1:** Clinical characteristics of the sample of 30 patients with unilateral definite Meniere’s disease who underwent 1.5 T and 3.0 T inner ear MRI.

Clinical variables			*n* (%)
Sex	Feminine		17 (56.7)
	Masculine		13 (43.3)
Age (years)	Mean (SD)	49.6 (12.5)	
	Median (min–max)	47 (22–73)	
Time onset of the Meniere disease	Mean (SD)	7.2 (7.9)	
	Median (min–max)	5.5 (1–33)	
Crises number in the last year	Mean (SD)	4.3 (4.2)	
	Median (min–max)	2.5 (1–15)	
Treatment	Betahistine		14 (46.7)
	Betahistine + other		14 (46.7)
	Other medications		2 (6.7)
Betahistine Dose	Without Betahistine		2 (6.7)
	Betahistine 24 mg bid		12 (40.0)
	Betahistine 48 mg bid		8 (26.7)
	Betahistine 48 mg trid		8 (26.7)
Phenotypes	1		13 (43.3)
	2		6 (20.0)
	3		0 (0.0)
	4		8 (26.7)
	5		3 (10.0)
Meniere disease staging	1		0 (0.0)
	2		4 (13.3)
	3		23 (76.7)
	4		3 (10.0)

### Intraobserver analysis for the presence of EH at MRI

The E1 found signs of EH in 26 (86.66%) patients in the 3 T MRI: 16 (53.33%) of which were bilateral, while the remaining 10 (left ear, 6; right ear, 4) were unilateral. Thus, a total of 42 ears (70%) had signs of MD. In the 1.5 T MR, E1 found signs of EH in 25 (83.33%) patients, being 9 (30%) in the left ear, 8 (26.66%) in the right ear, and 8 (26.66%) bilateral, totaling 33 (55%) affected ears.

The E2 found signs of EH in 25 (83.33%) patients in the 3 T MRI, 3 (10%) in the right ear, 6 (20%) in the left ear, and 16 (53.33%) bilateral, totaling 41 (68.33%) ears. In the 1.5 T MRI, E2 found signs of EH in 22 (73.33%) patients, 7 (23.33%) in the right ear, 7 (23.33%) in the left ear, and 8 (26 0.66%) bilateral, totaling 30 (50%) ears.

Regarding the location of EH at 3 T MRI, E1 observed CH in 18 (60%) of the SE and in 16 (53.3%) of the AE; VH was detected in 22 (73.33%) of the SE and in 13 (43.3%) of the AE. In the 1.5 T MRIs, E1 observed CH in 19 (63.33%) of the SE and in 21 (70%) of the AE; VH was seen in 20 (66.66%) of the SE and in 7 (23.33%) of the AE.

Regarding EH grade, cochlear and vestibular grade II EH were found simultaneously with 3.0 T MRI in seven patients, all had crisis in the last 6 months, 4 of them in the last 2 months.

In AE according to the clinical criteria, there were 16 (53.33%) cases with CH and 14 (46.66%) cases with VH at 3.0 T MRI, in CH there was a predominance of grade I. In VH there was also a predominance of EH grade I, 13 cases (92.85%). Only one case in AE had EH grade II cochlear and vestibular simultaneously.

Regarding the finding of bilateral EH by MRI, it was observed that the five patients (16.66%) with 10 or more years of disease were all classified as having bilateral EH by 3.0 T MRI.

There was no agreement between the findings of EH according to MRI of 3.0 T and the presence of clinical symptoms for both examiners (*p* > 0.05). In the 1.5 T MRI, there was low (but statistically significant) agreement between the presence of EH in the scans and the SE findings for both examiners (Table 2).

**Table 2:** Agreement between the findings of endolymphatic hydrops on MRI at 3.0 T and 1.5 T in relation to symptomatic and asymptomatic ears, according to the clinical criteria for patients with unilaterally defined Meniere’s disease.

	Hydrops side	Symptomatic ear		Total	kappa (*κ*) (CI 95%)	*p* value
		Right ear	Left ear			
		*n* (%)	*n* (%)			
Examiner 1						
3 T MRI	None	2 (15.4)	2 (11.8)	4 (13.3)	0.115	0.066
	Right	3 (23.1)*	1 (5.9)	4 (13.3)	(−0.007; 0.237)	
	Left	1 (7.7)	5 (29.4)*	6 (20.0)		
	Bilateral	7 (53.8)	9 (52.9)	16 (53.3)		
	Total	13	17	30		
1.5 T MRI	None	3 (23.1)	2 (11.8)	5 (16.7)	0.347	<0.001
	Right	8 (61.5)*	0	8 (26.7)	(0.167; 0.526)	
	Left	1 (7.7)	8 (47.1)*	9 (30.0)		
	Bilateral	1 (7.7)	7 (41.2)	8 (26.7)		
	Total					
Examiner 2						
3 T MRI	None	3 (23.1)	2 (11.8)	5 (16.7)	0.091	0.116
	Right	2 (15.4)*	1 (5.9)	3 (10.0)	(−0.020; 0.202)	
	Left	1 (7.7)	5 (29.4)*	6 (20.0)		
	Bilateral	7 (53.8)	9 (52.9)	16 (53.3)		
	Total					
1.5 T MRI	None	5 (38.5)	3 (17.6)	8 (26.7)	0.217	0.007
	Right	6 (46.2)*	1 (5.9)	7 (23.3)	(0.061; 0.374)	
	Left	1 (7.7)	6 (35.3)*	7 (23.3)		
	Bilateral	1 (7.7)	7 (41.2)	8 (26.7)		
	Total					

*Correspond to the ears in which the findings were concordant.

In the comparison between the findings obtained in the 3.0 T and 1.5 T MRI regarding the prediction of EH, a moderate and statistically significant agreement was observed between tests for both examiners (Table 3).

**Table 3:** Agreement between 3.0 T and 1.5 T MRI methods in relation to the finding of symptomatic ear analyzed by two examiners in patients with unilaterally defined Meniere’s disease.

1.5 T MRI	3 T-MRI				Total	kappa (*κ*) (CI95%)	*p* value
	None	Right ear	Left ear	Bilateral			
	*n* (%)	*n* (%)	*n* (%)	*n* (%)	*n* (%)		
Examiner 1							
None	2 (50.0)^*^	2 (50.0)	0	1 (6.3)	5 (16.7)	0.414	<0.001
Right ear	1 (25.0)	2 (50.0)^*^	0	5 (31.3)	8 (26.7)	(0.193–0.636)	
Left ear	0	0	6 (100.0)^*^	3 (18.8)	9 (30.0)		
Bilateral	1 (25.0)	0	0	7 (43.8)^*^	8 (26.7)		
Total	4 (100.0)	4 (100.0)	6 (100.0)	16 (100.0)	30 (100.0)		
Examiner 2							
None	3 (60.0)^*^	1 (33.3)	1 (16.7)	3 (18.8)	8 (26.7)	0.462	<0.001
Right ear	1 (20.0)	2 (66.7)^*^	0	4 (25.0)	7 (23.3)	(0.245–0.679)	
Left ear	1 (20.0)	0	5 (83.3)^*^	1 (6.3)	7 (23.3)		
Bilateral	0	0	0	8 (50.0)^*^	8 (26.7)		
Total	5 (100.0)	3 (100.0)	6 (100.0)	16 (100.0)^*^	30 (100.0)		

* Ears in which the clinical criteria and radiological findings were agreement.

### Interobserver analysis

The agreement between the examiners’ assessments in relation to the EH was high (0.844) (Table 4) for the 3 T MRI and substantial (0.645) for the 1.5 T (Table 5).

**Table 4:** Agreement between the two examiners regarding the findings of ears without and with endolymphatic hydrops at 3.0 T MRI in patients with unilaterally defined Meniere’s disease.

3T MRI	Hydrops side	Examiner 1					kappa (*κ*) (CI95%)	*p* value
		None	Right ear	Left ear	Bilateral	Total		
		*n* (%)	*n* (%)	*n* (%)	*n* (%)	*n* (%)		
Examiner 2	None	4 (100.0)^*^	1 (25.0)	0	0	5	0.844	<0.001
	Right ear	0	2 (50.0)^*^	0	1 (6.3)	3	(0.679; 1.008)	
	Left ear	0	0	6 (100.0)^*^	0	6		
	Bilateral	0	1 (25.0)	0	15 (93.7)^*^	6		
	Total	4 (100.0)	4 (100.0)	6 (100.0)	16 (100.0)	30		

* Correspond to the ears in which the findings were concordant.

**Table 5:** Agreement between the two examiners regarding the findings of ears without and with endolymphatic hydrops at 1.5 T MRI in patients with unilaterally defined Meniere’s disease.

1.5 T MRI	Hydrops side	Examiner 1				Total	kappa (*κ*) (CI95%)	*p* value
		None	Right ear	Left ear	Bilateral			
		*n* (%)	*n* (%)	*n* (%)	*n* (%)			
Examiner 2	None	4 (80.0)^*^	1 (12.5)	1 (11.1)	2 (25.0)	8	0.645	<0.001
	Right ear	0	6 (75.0)^*^	1 (11.1)	0	7	(0.438; 0.853)	
	Left ear	1 (20.0)	0	6 (66.7)^*^	0	77		
	Bilateral	0	1 (12.5)	1 (11.1)	6 (75.0)^*^	88		
	Total	5 (100.0)	8 (100.0)	9 (100.0)	8 (100.0)	30		

* Ears in which the clinical criteria and radiological findings were agreement.

In order to better observe the ability of resonance to detect the affected ear, we considered in Table 6 only those patients in whom the 3 T MRI detected as having unilateral HE and excluded those whose findings were absence of HE and bilateral HE. For these remaining patients, we observed agreement between the 3.0 T MRI for both examiners and the SE according to the clinical criterion. This time, the agreement between the findings was moderate for both, with kappa values of 0.583 for R1 and 0.500 for R2. And in relation to the findings of the 1.5 T MRI, the agreement was almost perfect (*k* = 0.0883) for the findings of R1 and substantial (*k* = 0.714) for R2. In the same table, we observed an accuracy greater than 77% for both exams and for both examiners (Table 6).

**Table 6:** Agreement and accuracy between the findings of unilateral endolymphatic hydrops on 3.0 T and 1.5 T MRI in relation to the symptomatic ear, according to clinical criteria, for both examiners, in patients with unilateral defined Meniere’s disease, excluding the findings on MRI of absent and bilateral endolymphatic hydrops.

Method	Symptomatic ear by clinical criteria		kappa	*p* value	Accuracy
	Right ear	Left ear	(95% CI)		
Examiner 1					
3 T MRI (*n* = 10)					
Right	3 (75.0)	1 (16.7)	0.583	0.065	80.0%
Left	1 (25.0)	5 (83.3)	(0.069; 1.098)		
Total	4 (100.0)	6 (100.0)			
1.5 MRI (*n* = 17)					
Right	8 (88.9)	0	0.883	<0.001	94.1%
Left	1 (11.1)	8 (100.0)	(0.661; 1.104)		
Total	9 (100.0)	8 (100.0)			
Examiner 2					
3 T MRI (*n* = 9)					
Right	2 (66.7)	1 (16.7)	0.500	0.134	77.8%
Left	1 (33.3)	5 (83.3)	(−0.100; 1.100)		
Total	3 (100.0)	6 (100.0)			
1.5 T MRI (*n* = 14)					
Right	6 (85.7)	1 (14.3)	0.714	0.008	85.8%
Ear	1 (14.3)	6 (85.7)	(0.348; 1.081)		
Total	7 (100.0)	7 (100.0)			

*Correspond to the ears in which the findings were concordant.

95% CI: confidence interval of 95%. The hatched cells correspond to the ears in which the findings were concordant.

### Analysis between the degree of EH at MRI and clinical aspects

We did not find significant correlations between any clinical variable or severity of hearing loss and the degree of CH and VH in the 3 T MRI (Table 7).

**Table 7:** Association between clinical variables of patients with unilateral defined Meniere’s disease and the finding of the degree of cochlear and vestibular endolymphatic hydrops on 3.0 T MRI.

Clinical parameters		Cochlear endolymphatic hydrops			*p* value	Vestibular endolymphatic hydrops			*p* value
		3 T MRI				3 T MRI			
		Grade 0	Grade 1	Grade 2		Grade 0	Grade 1	Grade 2	
Onset time of disease	Mean (SD)	6.6 (8.7)	8.1 (6.4)	7.1 (9.1)	0.522^1^	4.6 (2.0)	7.3 (6.3)	9.2 (11.8)	0.809^1^
	Median (Q1–Q3)	5 (1.5-6.5)	6 (3–10)	5 (2–6.5)		5.5 (3.5–6)	5 (3–9.5)	5 (1–7)	
Attacks in the last year	Mean (SD)	3.4 (3.9)	4.3 (3.6)	5.8 (5.3)	0.481^1^	1.6 (0.7)	5.5 (4.3)	5.1 (4.9)	0.116^1^
	Median (Q1–Q3)	2 (1–3)	4 (1–8)	3 (2–10)		1.5 (1–2)	5.5 (1–9)	3 (2–8)	
Months without attacks	Mean (SD)	4.2 (3.6)	4.9 (4.2)	3.1 (2.6)	0.472^1^	5.4 (3.1)	4.0 (4.1)	3.3 (3.2)	0.396^1^
	Median (Q1–Q3)	4.5 (0.5-7.5)	4 (1–10)	2.5 (0.6–6)		5.5 (3.5-7.5)	1 (1–7.5)	2 (0.2–6)	
DHI*	Mean (SD)	48.5 (21.4)	35.6 (19.3)	47.0 (26.8)	0.371^1^	43.8 (27.4)	47.2 (24.1)	39.8 (16.7)	0.846^1^
	Median (Q1–Q3)	47 (37–61)	33 (22–48)	45 (23–62)		37 (22–60)	47 (30–64)	43 (24–48)	
Phenotype *n* (%)	1	4 (33.3)	5 (50.0)	4 (50.0)	0.849^2^	2 (25.0)	6 (50.0)	5 (50.0)	0.445^2^
	2	2 (16.7)	2 (20.0)	2 (25.0)		1 (12.5)	2 (16.7)	3 (30.0)	
	4	4 (33.3)	3 (30.0)	1 (12.5)		3 (37.5)	4 (33.3)	1 (10.0)	
	5	2 (16.7)	0	1 (12.5)		2 (25.0)	0	1 (10.0)	

^1^Teste de Kruskal-Wallis; ^2^Teste exato de Fisher. *DHI = Dizziness handicap inventory.

### Image findings

**Figure 1:**
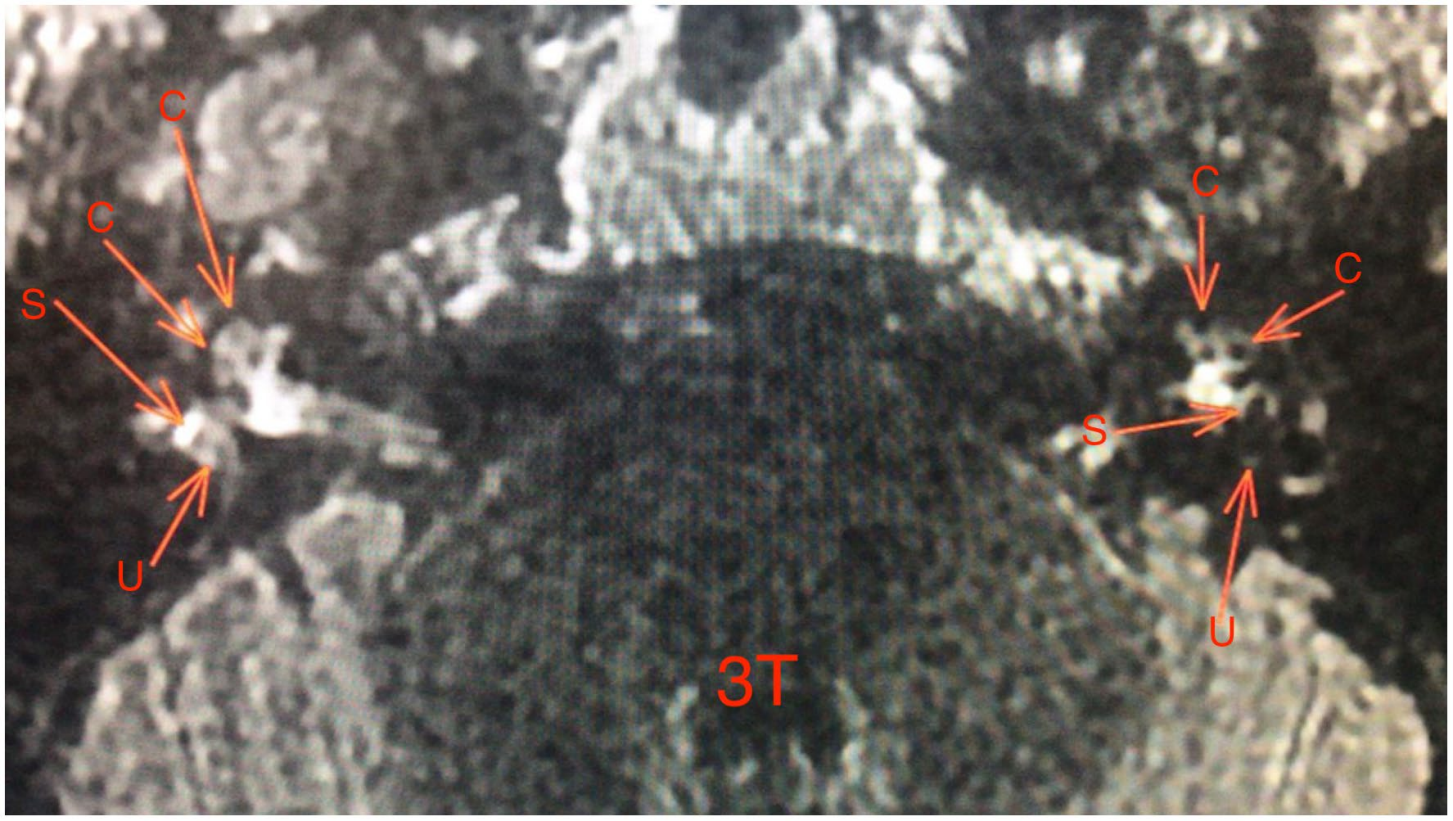
3 T MRI axial scan, sequence 3D-FLAIR, right ear with endolinphatic hydrops in cochlea and vestibule grade 0 and left ear with grade 2 of Barath in cochlea and vestibule.

**Figure 2:**
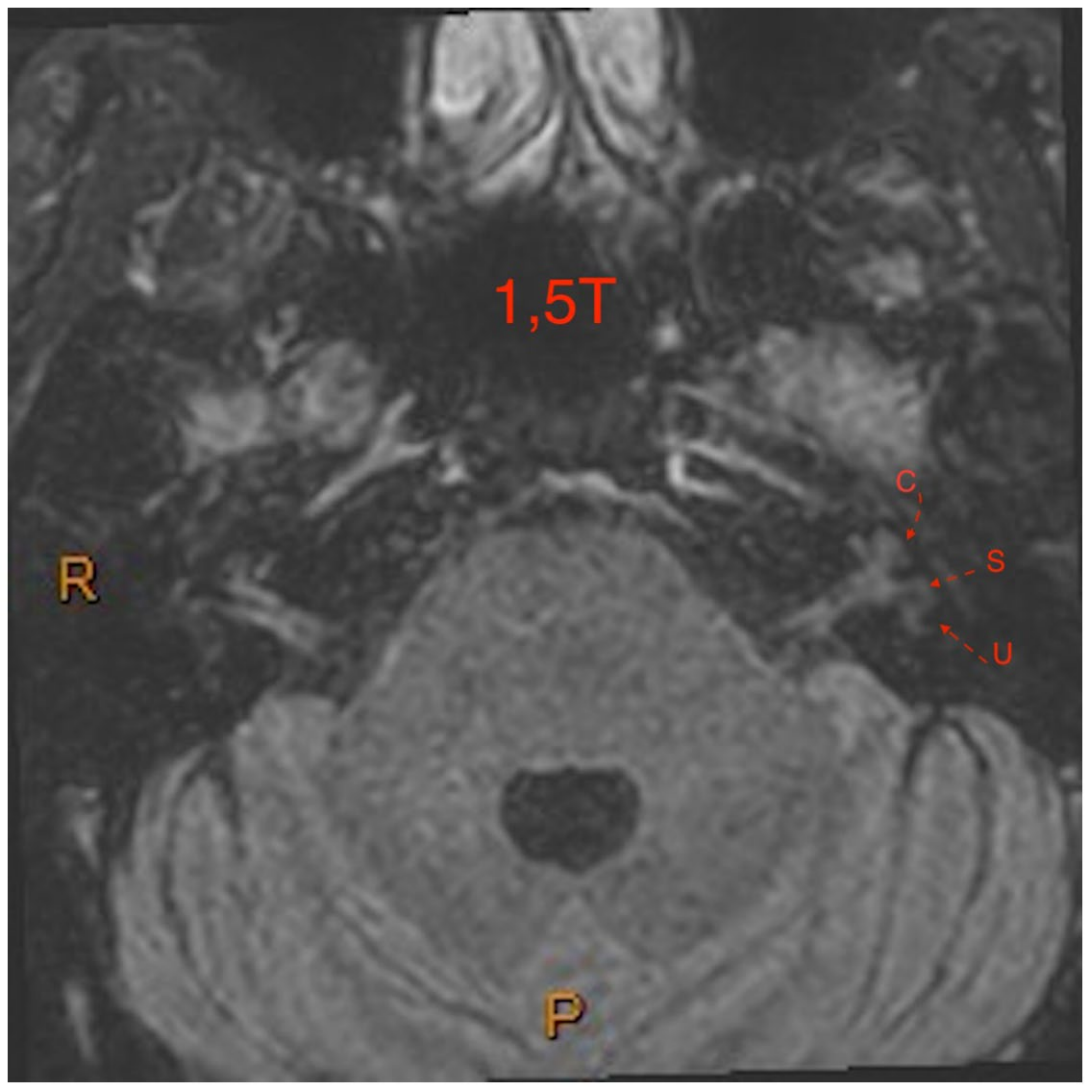
1.5 T MRI axial scan, sequence 3D-FLAIR, left ear with grade 0 of Barath in cochlea and vestibule.

**Figure 3:**
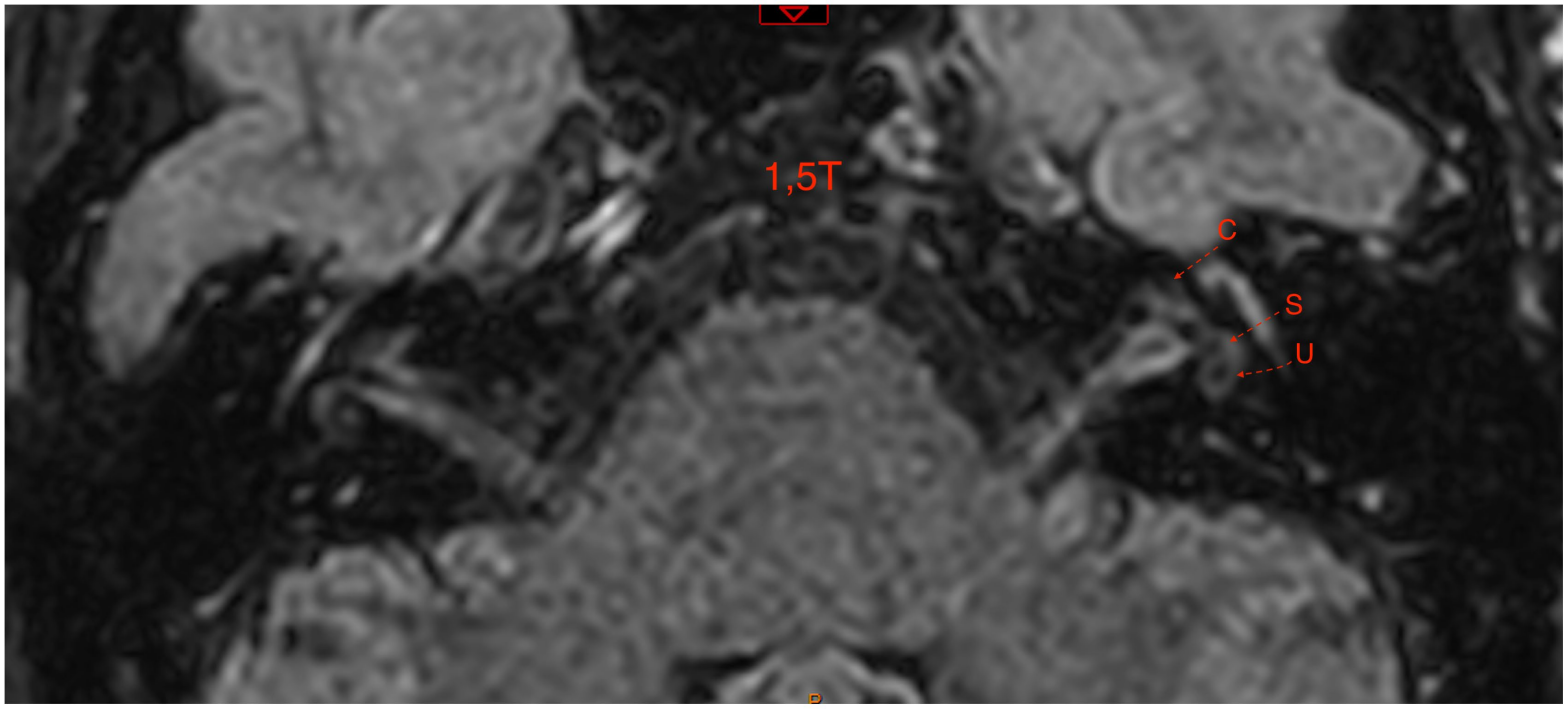
1.5 T MRI axial scan, sequence 3D-FLAIR, left ear with grade 1 of Barath in cochlea and vestibule.

**Figure 4:**
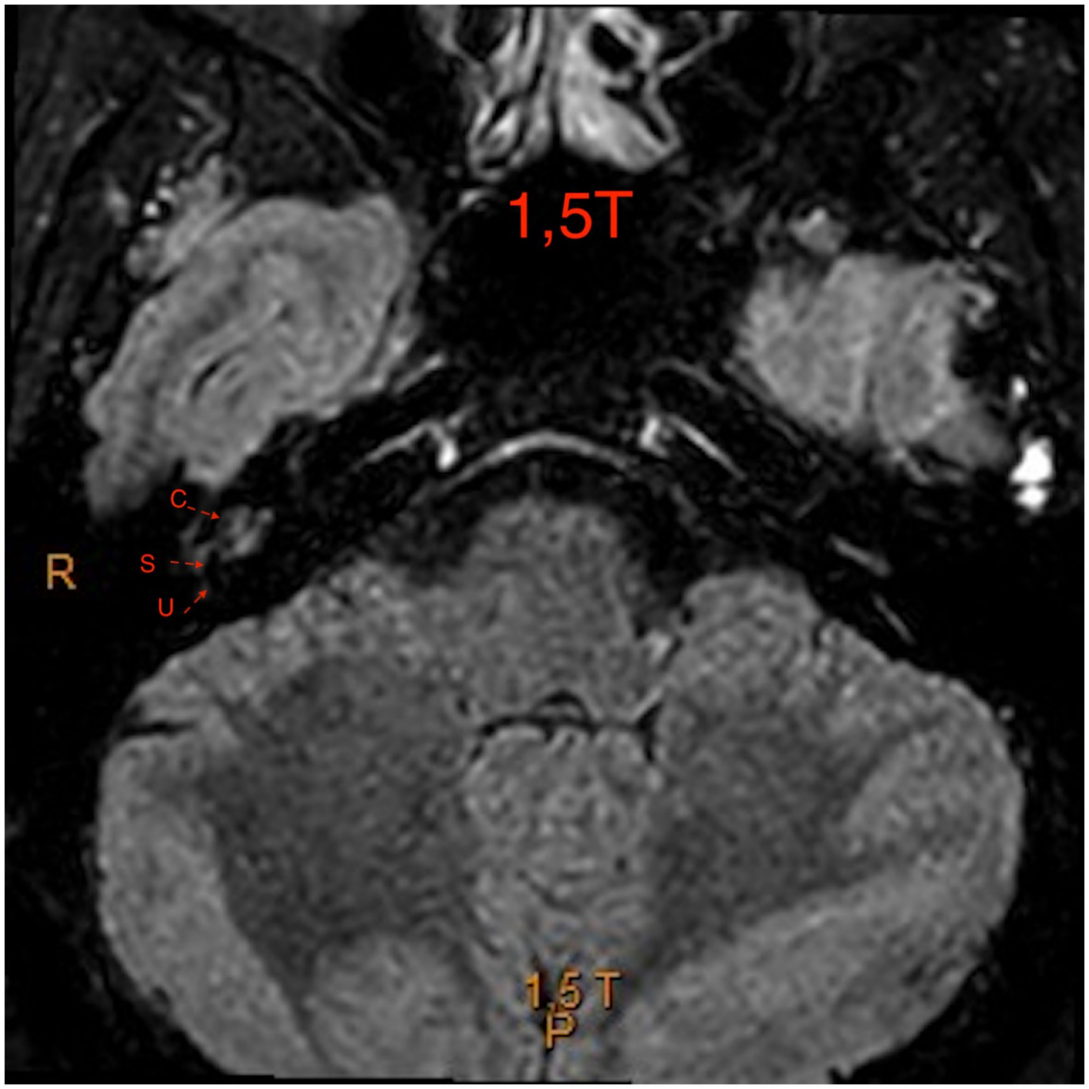
1.5 T MRI axial scan, sequence 3D-FLAIR, right ear with grade 2 of Bárath in cochlea and vestibule.

The figures are examples of images of four different patients. In Figure 1, the images are in 3 T and Figures 2–4, are images in 1.5 T, all of them in the 3D-FLAIR sequence, 4 h after IV GC injection.

## Discussion

The magnetic field intensity of MRI varies, with 1.5 T and 3 T being the most commonly used. The advantages of a larger field are: increased signal-to-noise ratio, which improves the temporal and/or spatial resolution of the image, with this ratio being approximately twice as high for 3 T; reduced acquisition time, which allows for a greater number of sequences; increased T1 relaxation time intensity and reduced T2 relaxation time by up to 10%. However, the disadvantage of a larger field is the increase in chemical shift artifacts, for example, causing spatial registration errors of fat and water in the image (13).

The improvement of imaging capture and sequence techniques, such as the use of the fast spin echo technique and time inversions, have allowed, for example, the suppression of the cerebrospinal fluid signal. The technique called FLAIR is a fast spin echo with IR with long time inversion, in these images zero is displayed in gray, the negative image is hypointense and the positive is hyperintense, this image is especially used in the study of the central nervous system (14).

For the study of temporal bone, the recommended protocol includes T1-weighted sequences without and with contrast, thin slices smaller than 3 mm and T2-weighted volumetric sequences with 1 mm slices, the so-called cisternographic sequence and which also receives different names according to the device used (FIESTA, SPACE, CISS, DRIVE among others) (15). However, even with the use of these sequences, the visualization of the ES remained a challenge and studies from the 2000s onwards began with image inversion techniques, which made it possible to visualize the ES.

Nakashima et al., carried out the first study that obtained images of the ES and its dilation in cases of EH. They evaluated four patients with MD, four patients with sudden deafness and one patient with severe SNHL. They used 3 T MRI with eight channels, in the sequences heavily T2-weighted 3D constructive interference in the steady state image (hT2-w 3D-CISS) and 3D-FLAIR (5).

Nakashima et al., observed that in the 3D-FLAIR sequence after IT injection of GD, although perilymph enhancement (PPI) occurred, the boundaries between the bone and ES were not evident, so they proposed short time inversions, the ideal shortening of the time inversion in 3D-FLAIR suppressed the signal from the perilymph containing the GC instead of increasing the signal from the endolymph without contrast (PEI), thus with IV GC administration the use of hT1-w 3D FLAIR with PEI and PPI acquisition ensured the visualization of HE. With the use of IT contrast, the 3D-real IR sequence was used, but when it was used with IV GC, they did not achieve the ideal amount of contrast in the perilymph. Therefore, the authors suggested using the subtraction of these PEI and PPI images to improve the enhancement of the perilymph, which they called the HYDROPS protocol (HYbriD of Reversed image Of Positive endolymph signal and native image of positive perilymph signal) (7). In 2014, the same authors proposed a modification of the HYDROPS protocol by HYDROPS 2 (hT2-w 3D-FLAIR) after 4 h of using IV GC. In this sequence, after using GC, the perilymph enhances and the bone and ES present a value close to zero (PPI). To better distinguish the bone limit from the ES, a short time inversion should be performed. This inversion removes the enhancement of the GD from the EP and starts to enhance the EE (PEI). Another sequence described that separates the EP images from EE and bone is the 3D-real IR, but it has only been used for GD IT use, as it shows a low concentration of GD in the perilymph (16).

The detection of affected ear on MD is based on the presence of various qualitative or semi-quantitative MRI descriptors and grading scales which define expansion of the ES. However, there is no universal consensus on which specific MRI features best distinguish affected ears (17).

In a systematic review, the variability of the articles was observed in relation to the classifications used to measure EH in the Nakashima, Barath, Beranaerts, Kahn. As well as the clinical variability of samples using probable, possible DM, sudden deafness, primary and secondary hydrops. It is difficult to compare studies and therefore the high variability in sensitivity (69–92%) of the MRI (18).

Baráth et al., found 95% sensitivity of 3 T MRI in detecting EH in patients with defined DM; they found a high correlation between the finding of EH and the diseased ear in these patients, however, they found 92% of CH in AE (12). Due to this apparent low specificity related to CH, Bernaerts et al., considering only HV in the evaluation of patients with DM, presented a sensitivity of 84.6% and a specificity of 93.6% (19). These two studies consider the qualitative evaluation of EE (12, 20).

In the current study, 3D-FLAIR sequence was used 4 h after IV GC injection.

In addition to the image acquisition protocol, another challenge is the parameters used to infer the widening of the endolymphatic spaces. More recent studies have attempted to observe which parameter is more specific for detecting EH and its degrees. Connor et al., carried out a retrospective study, examined consecutively 96 patients and 78 controls, forward stepwise logistic regression determined which combination of MRI descriptors would best predict MD ears, absent, enlarged or confluent saccules are the best predictors of MD. Incomplete visualization of the vestibular aqueduct added value to the diagnosis (18).

In the present study, it was observed that the finding of CH did not vary between SE and AE and was around 60%; on the other hand, VH was visualized more frequently in SE in 22 (73.33%) than in AE (43.3%). The findings were similar for 1.5 T MRI, with VH being found in 20 (66.66%) in SE and in 7 (23.33%) in AE. The findings of HV prevalence in patients with MD on MRI were similar to those of Okumura et al., who found 70% (14/20) HV in DM cases (19). And the same relationship of VH more prevalent in patients with MD and CH more prevalent in healthy individuals (controls) was seen by Conte et al. (17).

In the present study, both the 1.5 and 3 T MRI scans were able to identify EH in SEs of patients with MD. The majority of previous investigations used 3 T MRI scans to assess for EH in patients with MD. Some of these previous studies showed that all ears with definite MD showed EH on their MRIs; however, these studies were conducted in small cohorts of patients (4, 21–23). It is difficult to assess the sensitivity and specificity of the 3 T MRI to identify SE, as no other clinical or imaging test can be considered a “gold standard” to compare with the MRI findings and histologic comparison is not feasible. Nonetheless, some authors have used the terms sensitivity and specificity to describe imaging tests by comparing with clinical diagnosis. Baráth et al. found that the 3 T MRI has a 95% sensitivity to detect the presence of EH in SE (12). As it was shown that the presence of CH in MRI tests is not specific to MD, most authors assessed VH in isolation to evaluate for EH in the context of MD. Bernaerts et al., for example, reported that the presence of VH had a sensitivity of 84.6% and a specificity of 93.6% for diagnosing MD (20). Other authors, however, reported lower prevalence rates of VH in patients with MD, ranging from 48 to 50% (24, 25). Studies evaluating the 1.5 T MRI scan to identify EH are very scarce. Our results showed that, on 1.5 T MRI scans, EH was found in 83.3 and 73.3% of our definite MD cohort (E1 and E2, respectively). There are only two studies that used 1.5 T MRI to assess for the presence of EH. Grieve et al. found EH in 11 of 13 MD patients (84.61%) following intratympanic gadolinium contrast injection (6). Naganawa et al. observed some degree of EH in all 20 MD patients in at least one ear using intravenous gadolinium contrast (7).

Our results showed a significant positive correlation between the Baráth grade of EH and the number of acute episodes in the last year. The seven patients with grade 2 EH (CH and VH) had an episode in the last 6 months and were related to worse scores of DHI. Similarly, Shimono et al., found a trend toward more severe EH in more symptomatic cases (24).

Regarding the degree of NSHL according to clinical staging, no statistically significant association with the degrees of CH and VH was found in the 3.0 T MRI, results that agree with the findings by Fukuoka et al. (25). Some studies found an association between the degree of EH and the severity of HL; however, they used the mean of pure tone thresholds, which differs from the variable used in the current study, which was the degree of clinical staging.

Nakashima et al., thus suggested that the highest grade of EH on hydrops MRI is used for interpretation, which may not match the responsible site for pure tone average from 500, 1,000, 2,000, and 3,000 Hz (5). Young et al., suggested using sum of three low-frequency (125, 250, and 500 Hz) hearing levels >100 dB to predict positive CH. In contrast, that <100 dB, MRI should be postponed since small-sized EH may be ignored. However, human measurements for grading of EH are prone to variability. One may imagine a future artificial intelligence to calculate the volumes of endolymph and perilymph based on the contrast density gradient between the 2 (14).

Interestingly, EH was identified in some of the AE of patients included in our study (53% in the 3 T MRI). These findings are similar to those of House et al., who found that 55% of the AE from patients with MD later developed clinical MD (26). Other authors have also reported signs of EH in the AE of patients with MD in 3 T MRs, with the incidence ranging from 22% (27) to 65% (22). The presence of signs of EH in AE should not be interpreted as a false positive result: it is possible that the findings represent an early, asymptomatic stage of the disease. Indeed, EH has also been observed in asymptomatic healthy individuals, as shown by histopathological and imaging studies. Indeed, the literature shows that MD can frequently affect both ears, affecting 30–40% of all patients with MD (26).

Our 3 T MRI scans did not show a significant difference in the rates of CH between SE and AE (around 60%). Conversely, VH was observed more frequently in the SE (73%) than in AE (43%). The 1.5 T MRI scans showed similar findings regarding the prevalence of VH (SE, 66%; AE, 23%). The prevalence of VH in SE were similar to what described by Okumura et al. (70%) (19). Interestingly, Conte et al. also observed that CH tended to occur in AE, while the presence of VH had a stronger correlation with the SE (17).

Patients with phenotypes 1 and 2 of MD presented with EH in 100% of cases. Conversely, patients with phenotypes 4 and 5 did not present with signs of EH, even though all patients with phenotypes 4 and 5 met the proposed criteria for definite MD. To our knowledge, there are no studies in the literature dedicated to comparing MRI findings with MD phenotypes. Although we can only speculate on what could have caused these results, it is possible that patients who have those phenotypes might have distinct pathophysiological mechanisms and as compared with those of other groups, and therefore present atypical findings on the imaging scans.

A major goal of our study was to investigate the validity of the 1.5 T MRI protocol and to compare it with that of 3 T MRI. Although 3 T MRI for hydrops assessment is quite established and is gaining traction in clinical communities to study the presence of EH in atypical cases, 3 T scans are unavailable in some locations, especially in developing countries. Therefore, validating a 1.5 T MRI protocol to identify EH would increase global imaging accessibility, helping to increase clinical utilization of this diagnostic test to aid in diagnosis. The use of MRI in a clinical setting would, in this case, not only be limited to identifying EH in patients with suspected MD but would also be important for the purposes of differential diagnosis. Our results showed that the results obtained by two experienced examiners using both 3 T and 1.5 T MRI scans were similar, with both methods capable of providing similar imaging information.

The study is limited by its small sample size and the potential for selection bias. As only patients with definitive MD were included in our study cohort, there was a risk of observation bias. There was a high heterogeneity of clinical presentations and treatments among patients. Although analysis of a wide heterogeneous population is helpful for the purposes of assessing the effects of different variables and their influence in the MRI scans, it could theoretically have influenced some of the results. Another limitation was conducted in a single center, which may limit the generalizability of the findings. Another point can be the influence of different scanner types on image quality (GE and Philipps) and it would be the best to use one scanner type for the scans. Nonetheless, our results provide important data supporting the use of 1.5 T MRI scans to identify EH.

The use of MRI to identify HE is already occurring in clinical practice, could be considered an additional biomarker for detecting the dynamic status of Meniere’s disease (12). The most of the studies and protocols use 3 T devices, however 1.5 T devices are the most easily found and commonly used in clinical practice and their cost is lower than that of 3 T devices. Thus, verifying the accuracy of 1.5 T MRI could make the use of MRI in the DM more accessible to the majority of the population in the world.

## Conclusion

1.5 T and 3.0 T MRI images agreed regarding the findings of absence or presence of CH and VH. The degrees of CH and VH found at 3.0 T MRI in SE were not correlated with clinical aspects and the degree of disease staging in patients with unilateral definite MD.

## Data Availability

The datasets presented in this study can be found in online repositories. The names of the repository/repositories and accession number(s) can be found at: https://hdl.handle.net/11600/64239.

## References

[1] Lopez-Escamez JA Carey J Chung W Goebel JA Magnusson M Mandala M. Diagnostic criteria for Menière’s disease. J Vest Res. (2015) 25:1–7. doi: 10.3233/VES-150549 25882471

[2] Bisdorff AR Staab JP Newman-Toker DE. Overview of the international classification of vestibular disorders. Neurol Clin. (2015) 33:541–50. doi: 10.1016/j.ncl.2015.04.010, PMID: 26231270

[3] Magnan J Özgirgin ON Trabalzini F Lacour M Lopez-Escamez AL Magnusson M. European position statement on diagnosis, and treatment of Meniere’s disease. J Int Adv Otol. (2018) 14:317–21. doi: 10.5152/iao.2018.140818, PMID: 30256205 PMC6354459

[4] Hallpike CS Cairns H. Observations on the pathology of Meniere’s syndrome. J Laryngol Otol. (1938) 53:625–55. doi: 10.1017/S0022215100003947 PMC2076781 19991672

[5] Nakashima T Naganawa S Sugiura M Teranishi M Sone M Hayashi H. Visualization of endolymphatic hydrops in patients with Meniere’s disease. Laryngoscope. (2007) 117:415–20. doi: 10.1097/MLG.0b013e31802c300c 17279053

[6] Grieve SM Obholzer R Malitz N Gibson WP Parker GD. Imaging of endolymphatic hydrops in Meniere’s disease at 1.5T using phase-sensitive inversion recovery: (1) demonstration of feasibility and (2) overcoming the limitations of variable gadolinium absorption. Eur J Radiol. (2012) 81:331–8. doi: 10.1016/j.ejrad.2011.01.073, PMID: 21330087

[7] Naganawa S Yamazaki M Kawai H Bokura K Sone M Nakashima T. Imaging of Ménière's disease after intravenous administration of single-dose gadodiamide: utility of multiplication of MR cisternography and HYDROPS image. Magn Reson Med Sci. (2013) 12:63–8. doi: 10.2463/mrms.2012-0027 23474961

[8] Han Y Zhang J Lui L Gao X Wu L Du X. The diagnostic value of 1.5T versus 3.0T magnetic resonance imaging intratympanic gadolinium inner ear enhancement in patients with Meniere’s disease. J Int Adv Otol. (2022) 18:388–91. doi: 10.5152/iao.2022.21496, PMID: 36063094 PMC9524368

[9] Naganawa S Yamazaki M Kawai H Bokura K Sone M Nakashima T. Imaging of Ménière's disease by subtraction of MR cisternography from positive perilymph image. Magn Reson Med Sci. (2012) 11:303–9. doi: 10.2463/mrms.11.303 23269018

[10] Surgery N Monsell M Balkanni TA. Committee on hearing and equilibrium guidelines for the diagnosis and evaluation of therapy in menière’s disease. american academy of otolaryngology - head and neck foundation, inc. otolaryngol head neck Surg. J Am Acad Otolaryngol Head Neck Surg. (1995) 113:181–5. 10.1016/S0194-5998(95)70102-8 7675476

[11] Frejo L Soto-Varela A Santos-Perez S Aran I Batuecas-Caletrio A Perez-Guillen V. Clinical subgroups in bilateral Meniere disease. Front Neurol. (2016) 7:1–10. doi: 10.3389/fneur.2016.00182 27822199 PMC5075646

[12] Baráth K Schuknecht S Naldi M Schrepfer T Bockisch CJ Hegemann SCA. Detection and grading of endolymphatic hydrops in Menière disease using MR imaging. Am J Neuroradiol. (2014) 35:1387–92. doi: 10.3174/ajnr.A3856, PMID: 24524921 PMC7966587

[13] Runge VM Nitz WR Heverhagen JT. The physics of clinical MR taught through images. 4th ed. New York: Thieme (2009).

[14] Young YH Lin KT. Potential application of hydrops MR imaging: a systematic review. J Otolaryngol Head Neck Surg. (2024) 53:19160216241250350. doi: 10.1177/19160216241250350, PMID: 38888936 PMC11098000

[15] Paes Junior AJ Haetinger RG. Cabeça e Pescoço In: Série do Colégio Brasileiro de Radiologia e Diagnóstico por Imagem. 1st ed. Rio de Janeiro: Elsevier (2017)

[16] Naganawa S Yamazaki M Kawai H Bokura K Tatsuo I Sone M. RM imaging of Ménierè's disease after combined intratympanic and intravenous injection of gadolinium using HYDROPS2. Magn Reson Med Sci. (2014) 13:133–7. doi: 10.2463/mrms.2013-0061, PMID: 24769636

[17] Conte G Lo Russo FM Calloni SF Sina C Barozzi S di Berardino F. MR imaging of endolymphatic hydrops in Ménière’s disease: not all that glitters is gold. Acta Otorhinolaryngol Ital. (2018) 38:369–76. doi: 10.14639/0392-100X-1986, PMID: 30197428 PMC6146579

[18] Connor S Pai I Touska P McElroy S Ourselin S Hajnal JV. Assessing the optimal MRI descriptors to diagnose Ménière's disease and the added value of analysing the vestibular aqueduct. Eur Radiol. (2024) 34:6060–71. doi: 10.1007/s00330-024-10587-w, PMID: 38326448 PMC11364795

[19] Okumura T Imai T Takimoto Y Takeda N Kitahara T Uno A. Assessment of endolymphatic hydrops and otolith function in patients with Ménière’s disease. Eur Arch Otorrinolaringol. (2016) 274:1413–21. doi: 10.1007/s00405-016-4418-2 27942898

[20] Bernaerts A Vanspauwen R Blaivie C van Dinther J Zarowski A Wuyts FL. The value of four stage vestibular hydrops grading and asymmetric perilymphatic enhancement in the diagnosis of Menière’s disease on MRI. Neuroradiology. (2019) 61:421–9. doi: 10.1007/s00234-019-02155-7, PMID: 30719545 PMC6431299

[21] Wu Q Dai C Zhao M Sha Y. The correlation between symptoms of definite Meniere’s disease and endolymphatic hydrops visualized by magnetic resonance imaging. Laryngoscope. (2016) 126:974–9. doi: 10.1002/lary.25576 26333096

[22] Suzuki H Teranishi M Sone M Yamazaki M Naganawa S Nakashima T. Contrast enhancement of the inner ear after intravenous administration of a standard or double dose of gadolinium contrast agents. Acta Otolaryngol. (2011) 131:1025–31. doi: 10.3109/00016489.2011.598552 21732744

[23] Attyé A Eliezer M Galloux A Pietras J Tropres I Schmerber S. Endolymphatic hydrops imaging: differential diagnosis in patients with Meniere disease symptoms. Diagn Interv Imaging. (2017) 98:699–706. doi: 10.1016/j.diii.2017.06.002, PMID: 28645678

[24] Shimono M Teranishi M Yoshida T Kato M Sano R Otake H. Endolymphatic hydrops revealed by magnetic resonance imaging in patients with acute low-tone sensorineural hearing loss. Otol Neurotol. (2013) 34:1241–6. doi: 10.1097/MAO.0b013e3182990e81, PMID: 23921924

[25] Fukuoka H Takumi Y Tsukada K Miyagawa M Oguchi T Ueda H. Comparison of the diagnostic value of 3 T MRI after intratympanic injection of GBCA, electrocochleography, and the glycerol test in patients with Meniere’s disease. Acta Otolaryngol. (2012) 132:141–5. doi: 10.3109/00016489.2011.635383, PMID: 22201289 PMC3490481

[26] House JW Doherty JK Fisher LM Derebery MJ Berliner KI. Meniere’s disease: prevalence of contralateral ear involvement. Otol Neurotol. (2006) 27:355–61. doi: 10.1097/00129492-200604000-00011, PMID: 16639274

[27] Tanigawa T Tamaki T Yamamuro O Tanaka H Nonoyama H Shiga A. Visualization of endolymphatic hydrops after administration of a standard dose of an intravenous gadolinium-based contrast agent. Acta Otolaryngol. (2011) 131:596–601. doi: 10.3109/00016489.2010.548402, PMID: 21351819

